# Validation of Instantaneous Respiratory Rate Using Reflectance PPG from Different Body Positions

**DOI:** 10.3390/s18113705

**Published:** 2018-10-31

**Authors:** Delaram Jarchi, Dario Salvi, Lionel Tarassenko, David A. Clifton

**Affiliations:** Department of Engineering Science, Institute of Biomedical Engineering, University of Oxford, Oxford OX3 7DQ, UK; dario.salvi@eng.ox.ac.uk (D.S.); lionel.tarassenko@eng.ox.ac.uk (L.T.); davidc@robots.ox.ac.uk (D.A.C.)

**Keywords:** respiratory rate, PPG, wavelet synchrosqueezed transform, modulation

## Abstract

Respiratory rate (RR) is a key parameter used in healthcare for monitoring and predicting patient deterioration. However, continuous and automatic estimation of this parameter from wearable sensors is still a challenging task. Various methods have been proposed to estimate RR from wearable sensors using windowed segments of the data; e.g., often using a minimum of 32 s. Little research has been reported in the literature concerning the instantaneous detection of respiratory rate from such sources. In this paper, we develop and evaluate a method to estimate instantaneous respiratory rate (IRR) from body-worn reflectance photoplethysmography (PPG) sensors. The proposed method relies on a nonlinear time-frequency representation, termed the wavelet synchrosqueezed transform (WSST). We apply the latter to derived modulations of the PPG that arise from the act of breathing.We validate the proposed algorithm using (i) a custom device with a PPG probe placed on various body positions and (ii) a commercial wrist-worn device (WaveletHealth Inc., Mountain View, CA, USA). Comparator reference data were obtained via a thermocouple placed under the nostrils, providing ground-truth information concerning respiration cycles. Tracking instantaneous frequencies was performed in the joint time-frequency spectrum of the (4 Hz re-sampled) respiratory-induced modulation using the WSST, from data obtained from 10 healthy subjects. The estimated instantaneous respiratory rates have shown to be highly correlated with breath-by-breath variations derived from the reference signals. The proposed method produced more accurate results compared to averaged RR obtained using 32 s windows investigated with overlap between successive windows of (i) zero and (ii) 28 s. For a set of five healthy subjects, the averaged similarity between reference RR and instantaneous RR, given by the longest common subsequence (LCSS) algorithm, was calculated as 0.69; this compares with averaged similarity of 0.49 using 32 s windows with 28 s overlap between successive windows. The results provide insight into estimation of IRR and show that upper body positions produced PPG signals from which a better respiration signal was extracted than for other body locations.

## 1. Introduction

Continuous monitoring of at high-risk hospital patients is a crucial step to improving patient outcomes by identifying physiological deterioration in patients, and informing clinicians [1]. Due to a high patient-to-nurse ratio in many healthcare settings, poor patient outcomes can occur when physiological deterioration goes unseen by clinical staff.

Wearable technologies, such as wrist-worn reflectance photoplethysmography (PPG) sensors, can be used to monitor physiological parameters such as heart rate, blood oxygen level (SpO${}_{2}$), and respiratory rate in a noninvasive manner. However, no such wearable technology is used at scale in healthcare practice. Although wearable technology has the potential to transform recognition of patients at risk of deterioration, current systems are less unobtrusive and not fully wearable (e.g., electrocardigraphy (ECG) electrodes, PPG sensors with finger probes, etc.). Additionally, they are used for averaged measurements over a long interval of time, such as a minute, and do not typically offer instantaneous values. Finally, they do not acquire and process the signals in a sufficiently reliable way for continuous monitoring in clinical or home environments, as would be required to identify many types of physiological deterioration. The primary reason that wearable sensors are not used at scale in clinical practice is that there is a lack of accuracy in the estimates of the vital signs that they produce. Often, this lack of accuracy is due to motion artefact that affects the recorded signals.

Of the commonly-acquired vital signs, the respiratory rate is one of the most important for measuring if physiological deterioration is to be identified [2,3]. The literature on the importance of measuring respiratory rate in clinical settings is substantial; for example, an observational study of 1025 emergency patients found that a respiratory rate of greater than 20 breaths per min (bpm) was directly related to cardiopulmonary arrest within 72 h and patient death within 30 days [4]. In another observational study of 1695 acute medical admissions, a mean respiratory rate of 27 bpm was related to cardiopulmonary arrest, intensive care admission, or death within 24 h [5]. It has been reported in various studies that abnormal respiratory rate is a highly informative indication for forthcoming adverse events [2,5,6,7,8].

To estimate respiratory rate, the ECG, PPG, and acceleration signals have been used in various research studies [9,10,11,12,13,14,15,16,17,18,19] among many others. While various methods in the literature have used windowed segments of the PPG/ECG signals, very few methods have investigated the instantaneous respiratory rate (IRR) from ECG [20,21] or PPG [22,23]. Recently-described methods have applied singular spectrum analysis (SSA) [24], or nonlinear time-frequency analysis; examples of the latter include the continuous wavelet transform (CWT) [25], the Hilbert transform [26], and variable-frequency complex demodulation [27,28]. In this work described by this paper, we address the estimation of instantaneous RR from reflectance PPG signals rather than using ECG signals (which usually require the application of electrodes and can cause discomfort). We demonstrate that, by using averaged estimated RR as it typical for most existing algorithms, variations in RR could be lost. These subtle variations can be tracked using the proposed method.

A key component of our proposed method is based on the wavelet synchrosqueezed transform (WSST) which has been used in [22] to estimate instantaneous respiratory rate from the publicly-available CapnoBase dataset applied to unfiltered PPG signals.

Novelty of the proposed work includes the use of the WSST in a non-standard manner, as will be outlined in the methodological description below, with the effect of improving time-frequency estimation with respect to existing methods. Additionally, we demonstrate the efficacy of our method using PPG signals recorded from various body positions—this represents a scenario that is close to the target application in clinical practice, whereby wristworn wearable sensors would be used for ambulatory patients. Existing analyses are typically restricted to analysis of the publicly-available CapnoBase dataset, in which patients are in a highly controlled, and non-ambulatory, environment.

**Figure 1:**
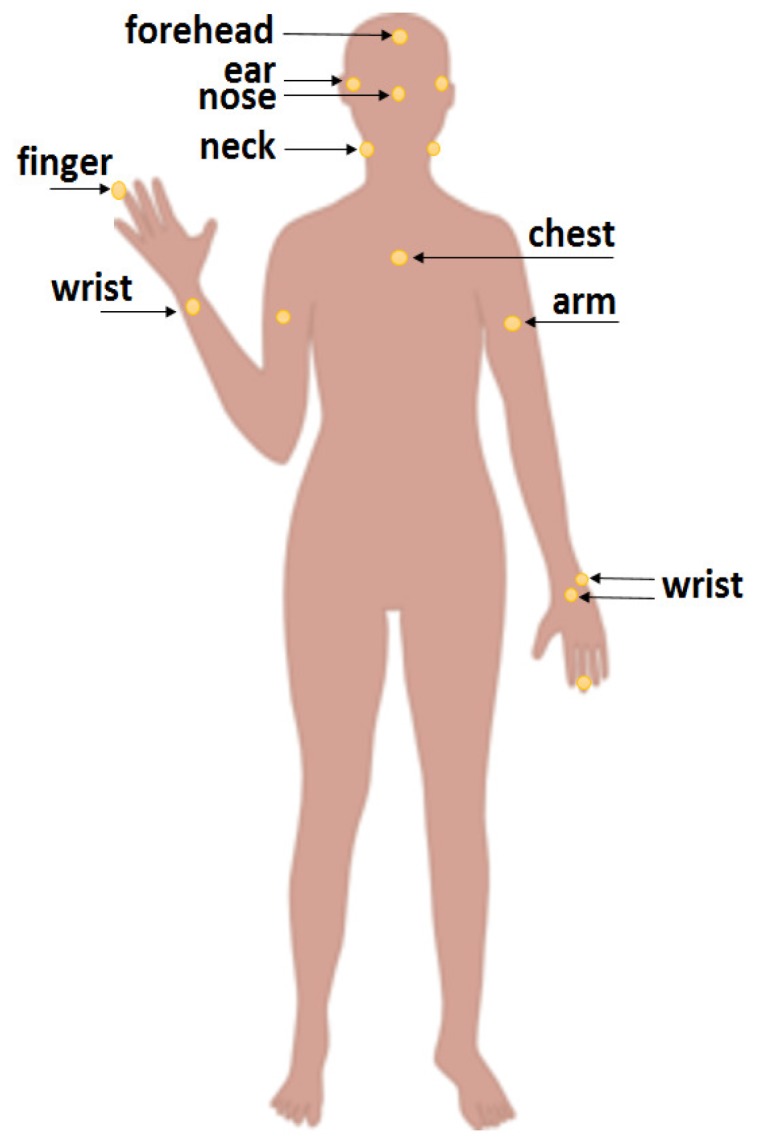
The PPG signals are recorded from various body positions using a custom device. For the wrist, three positions are considered such as the wrist-end of the ulna bone, and the centre of the wrist (front and rear positions).

**Figure 2:**
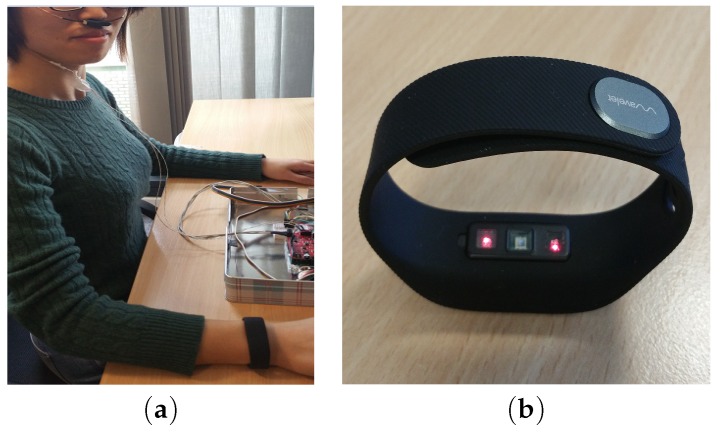
(**a**) A subject wearing the commercial Wavelet Health sensor. A thermocouple is used to provide ground-truth data for breath-by-breath respiration. (**b**) The Wavelet Health sensor.

The paper is organised as follows. Section 2 describes the proposed platform to estimate instantaneous respiratory rate. This section briefly explains the algorithms used to derive instantaneous RR including WSST, SSA, among others. In Section 3, we present results obtained for estimated IRR using two different datasets. Section 4 concludes the paper by discussing the main contributions of the paper, the limitations of the study, and potential future work. The main contributions of this work include:

- Proposing a new method to estimate IRR from reflectance PPG that is suitable for use with noninvasive, wristworn reflectance PPG sensors; and
- Validating the algorithm using a custom-made PPG device on various body positions (Figure 1), using a commercial wrist-worn sensor and a thermocouple for comparator, ground-truth data (Figure 2)

## 2. Methods

We first introduce the concept of respiration-induced modulations of the PPG, followed by explanations of (i) the method to derive respiratory-induced modulation from the PPG, (ii) the estimation of average RR, and (iii) the estimation of IRR. The latter describes the two main techniques (SSA and WSST) used in this paper to estimate IRR.

### 2.1. Respiratory-Induced Modulations

There are three important respiratory-induced modulations in PPG: intensity, amplitude, and frequency variations.

Intensity modulation (IM) is related to variation in intrathoracic pressure leading to a change in the baseline of perfusion [29]. IM is defined to be the variation in amplitude of the time-series of peak amplitudes of the PPG.

Frequency modulation (FM) is defined to be the change in the instantaneous heart rate during the respiratory cycle, also known as respiratory-sinus arrhythmia, which arises via regulation by the vagal nerve. FM is derived using the difference in the timing of consecutive pulse peaks in the PPG (which correspond to instantaneous heart rate).

Amplitude modulation (AM) is related to changes in cardiac output associated with the quantity of refill in the vessels at the periphery [29]. AM is defined to be the difference in peak amplitudes of consecutive peaks and troughs, effectively resulting in a time-series of the height of the PPG pulse.

### 2.2. Estimation of Average RR

Noting that the purpose of this paper is to address IRR estimation, we will consider average RR (taken over a window of 32 s duration) as a comparator. Average RR is estimated by first using SSA (which is defined at the end of this methodology section) to denoise the acquired PPG signal, and to remove its trend, such that detection of pulse peaks is straightforward, and for subsequent derivation of the respiratory-induced modulations (IM, AM, and FM) and application of spectral based methods. We note that, for simplicity, the PPG baseline has been removed by the use of SSA, and therefore only pulse peaks are used to generate AM and FM time-series.

We have chosen not to consider the use of IM in the work described by this paper, since preliminary implementation using IM time-series did not lead to reliable estimates of IRR. This is discussed later in the future work section.

Estimation of average RR (i.e., non-instantaneous RR) has been performed in various studies from PPG or ECG signals; for a detailed review, we refer the reader to [17]. One commonly-employed technique used for this latter task is based on autoregressive (AR) spectral analysis [30]. In this work, we also choose to estimate average RR by averaging the IRR estimates that we obtain as described below. Empirical investigation, not described here for brevity, demonstrate close similarity between average RR obtained via (i) averaging the IRR and (ii) via conventional AR-based methods. The considerable literature on the latter is not replicated here, where our concern is primarily the estimation of IRR.

### 2.3. Proposed Method for Estimation of IRR

Our method described in this section is based on the application of time-frequency analysis to the extracted modulations (AM, FM). Here, we will use the WSST transform (described in detail below) to capture instantaneous frequencies which are expected to be highly correlated with breath-by-breath variations.

**Figure 3:**
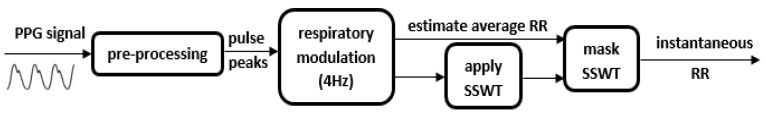
Diagram of the proposed algorithm to estimate IRR from wristworn PPG. The pre-processing stage includes the SSA algorithm which helps to better detect pulse peaks. Masking the time-frequency spectrum given by the WSST transform is crucial in identifying the appropriate band of respiratory frequencies, which will vary from patient to patient, and for changing health status of the individual subject.

As shown in Figure 3, the input PPG (acquired via red or infrared wavelengths of pulse oximeter) is first pre-processed. As mentioned earlier, this is performed by the SSA algorithm [31,32], from which respiratory modulations are extracted. As shown in the figure, we then apply our WSST-based approach as will be described in the next section.

### 2.4. Denoising via Singular Spectrum Analysis

The SSA algorithm has shown to be highly useful for denoising in various applications such as motion [32], heart rate [31], and respiration analysis [19]. The input to the SSA algorithm is the time-series and a value for the embedding dimension. Using the embedding dimension, the input time-series is converted into a matrix (called the trajectory matrix). Then, eigen-decomposition using singular value decomposition (SVD) is applied to the trajectory matrix. Based on the resulting eigenvalues, a number of eigenvectors and associated eigenvalues can be grouped to form an elementary matrix. This matrix will be transformed into a new reconstructed time-series using diagonal averaging. Based on the requirement of the target application, there are various ways to select the appropriate set of eigenvectors and associated eigenvalues in the reconstruction stage.

### 2.5. IRR Estimation via the Wavelet Synchrosqueezed Transform

The WSST is often used in the context of auditory signal processing [33]. WSST is based on time-frequency reassignment to enhance the time-frequency spectral representation of the signal [34], and comprises three steps:

*Step 1*: The continuous wavelet transform (CWT) is applied to the input signal s:

(1) $$W_{s}\left(a,b\right)=\int s\left(t\right)a^{-1/2}\bar{\psi (\frac{t-b}{a})}dt$$

where ψ is the selected mother wavelet, *t* is the time index, *a* is the wavelet scale, and *b* is the position parameter. The mother wavelet needs to be selected so that it is concentrated on the positive frequency axis; i.e., $\hat{\psi }\left(\varepsilon \right)=0$ for ε<0. Suppose we have a purely harmonic signal s(t)=Acos(ωt). Then, based on Plancherel’s theorem, the continuous WT can be re-written as:

(2) $$\begin{array}{cc} W_{s}\left(a,b\right) & =\int s\left(t\right)a^{-1/2}\bar{\psi (\frac{t-b}{a})}dt\\ & =\frac{1}{2\pi }\int \hat{s}\left(\varepsilon \right)a^{1/2}\bar{\hat{\psi }\left(a\varepsilon \right)}e^{ib\varepsilon }d\varepsilon \\ & =\frac{A}{4\pi }\int \left[\delta \left(\varepsilon -\omega \right)+\delta \left(\varepsilon +\omega \right)\right]a^{1/2}\bar{\hat{\psi }\left(a\varepsilon \right)}e^{ib\varepsilon }d\varepsilon \\ & =\frac{A}{4\pi }a^{1/2}\bar{\hat{\psi }\left(a\omega \right)}e^{ib\omega } \end{array}$$

In the case that $\hat{\psi }\left(\varepsilon \right)$ is concentrated around $\varepsilon =\omega_{0}$, then $W_{s}\left(a,b\right)$ will be concentrated around $a=\omega_{0}/\omega$, spreading out over a region around the horizontal line $a=\omega_{0}/\omega$. If $\omega =\omega_{0}/a$ is close to (but not equal to) the actual instantaneous frequency, then there will be some non-zero energy for $W_{s}\left(a,b\right)$. The concept of synchrosqueezing is to remove all of this energy from ω and reassign the frequency locations closer to the actual instantaneous frequency.

*Step 2*: In this step, the instantaneous frequency is estimated. The candidate instantaneous frequencies (ω(a,b)) can be obtained by the following equation, for which $W_{s}\left(a,b\right)\neq 0$:

(3) $$\omega \left(a,b\right)=-i{\left(W_{s}\left(a,b\right)\right)}^{-1}\frac{\partial }{\partial b}W_{s}\left(a,b\right)$$

In the case of having a purely harmonic signal s(t)=Acos(ωt), using Equation (2), ω(a,b) will immediately retrieve ω as required [33]. It is worth noting that the candidate instantaneous frequencies obtained from Equation (3) contain essential information to recover actual frequencies in the next step.

*Step 3*: In this step, the time domain is mapped into the time-frequency domain based on (b,a)⇒(b,ω(a,b)), which is synchrosqueezing. Each value of $W_{s}\left(a,b\right)$ is re-allocated into $T_{s}\left(\omega_{l},b\right)$, having $\omega_{l}$ as the closest frequency to the original point ω(a,b):

(4) $$T_{s}\left(\omega_{l},b\right)={\left(\Delta \omega \right)}^{-1}\sum_{a_{k}:\left|\omega \left(a_{k},b\right)-\omega_{l}\right|\leq \Delta \omega /2}W_{s}\left(a_{k},b\right)a_{k}^{-3/2}{\left(\Delta a\right)}_{k}$$

where $T_{s}\left(\omega_{l},b\right)$ is the synchrosqueezed transform at the centres $\omega_{l}$ of successive frequency bins, Δω represents the width of those frequency bins $\left[\omega_{l}-\frac{1}{2}\Delta \omega ,\omega_{l}+\frac{1}{2}\Delta \omega \right]$, $\Delta \omega =\omega_{l}-\omega_{l-1}$ and ${\left(\Delta a\right)}_{k}=a_{k}-a_{k-1}$. Therefore, at each fixed time point *b*, first the reassignment frequencies are estimated using Equation (3) for all scales. Then, for each desired instantaneous frequency of $\omega_{l}$, the value of $T_{s}\left(\omega_{l},b\right)$ has been calculated by summing all $W_{s}\left(a,b\right)$ where the distance of the reassigned frequency assignment ω(a,b) and $\omega_{l}$ is within a specified bin width (Δω/2).

In [33], it has been shown that following the synchronosqueezing stage, the original signal can be analytically reconstructed. Overall, $T_{s}\left(\omega_{l},b\right)$ is expected to be more sparse than $W_{s}\left(a,b\right)$ from the wavelet transform and is concentrated more sharply around actual instantaneous frequencies of the original signal. Therefore, by applying WSST transform into a desired signal (s(t)), instead of considering $W_{s}\left(a,b\right)$ (Equation (1)), we use $T_{s}\left(\omega_{l},b\right)$ (Equation (4)) to obtain the time-frequency spectrum of the input signal energy where time and frequency information are stored in *b* and $\omega_{l}$, respectively. To obtain $T_{s}\left(\omega_{l},b\right)$ for a desired input s(t), Equations (1), (3) and (4) must be applied.

Algorithm 1 describes how we apply SSA and the WSST for the estimation of IRR, which we here describe in detail.

As noted earlier, the SSA algorithm first defines a trajectory matrix; SVD is then applied to decompose it into eigenvectors and their associated eigenvalues. The eigenvectors with the largest associated eigenvalue (corresponding to the trend) and all eigenvalues below a predefined threshold are excluded; the remaining eigenvectors with associated eigenvalues are grouped to reconstruct an elementary matrix, which is the denoised and detrended signal.

**Algorithm 1** Estimation of Instantaneous RR from PPG signal.
**Input PPG**
p(t) ← red or infrared PPG
**Remove Base-line wander using SSA**
X ← trajectory-matrix [p(t), *M*], M, embedding dimension[U,Λ,V] ← Apply SVD onto X$\hat{X}=\sum_{j=2,3,...,L}\left[\sqrt{\lambda_{j}}u_{j}v_{j}^{T}\right]$, selection of eigenvectors L≤M$\hat{p}\left(t\right)$ ← diagonal-averaging$\left(\hat{X}\right)$, signal reconstruction
**Detect pulse peaks of the $\hat{p}\left(t\right)$**

**Estimate Respiratory induced modulation**
amplitude modulation (AM) or frequency modulation (FM)r(t)← Re-sample the modulation into a 4 Hz signal
**Estimate average Respiratory Rate (RR)**
$r_{a}\leftarrow$ average RR using Hilbert spectrum or AR-spectral method
**Apply SSWT to the 4 Hz modulation**
$\left[f,W_{ssw}\right]=SSWT\left(r\left(t\right)\right)$, replace s(t) by r(t) in Equation (1), implement Equations (3) and (4)returns f, instantaneous frequencies $\left(\omega_{l}\right)$and $W_{ssw}$, signal energy in time and frequency plan, ($i.e.,\;T_{r}(\omega_{l},b$))Mask SSWT spectrum using averaged RR
$\left(t_{i},i\right)=max\left[W_{ssw}\left(t\;:\;t\;+\;\delta t\;-\;1,r_{f}\;-\;\omega \;:\;r_{f}\;+\;\omega \right)\right]$
$r_{f}$ is associated respiratory frequency from average RR ($r_{a}$)δt, time-window size e.g., 32 s, ω, frequency-window size$t_{i}$, time indices, i, maximum-energy frequency indicesFind instantaneous frequencies $f_{ins}$
$f_{ins}\left(t_{i}\right)=f\left(i\right)$
**Estimate Instantaneous RR** in terms of breaths per minute ($f_{ins}\times 60$)

Pulse peaks are straightforwardly identified using standard peak-detection methods, yielding the AM and FM signals; the latter are then resampled at 4 Hz, which is a standard step in the literature [29,35,36,37,38] corresponding to a Nyquist frequency above that associated with the highest clinically-relevant value of respiratory rate. The normal respiratory frequency range is ≈[0.1 0.5] Hz that corresponds to 6–30 bpm. In [29], a conservative frequency range of [0.067 1.08] Hz relating to 4–65 bpm has been considered. Re-sampling the respiratory modulation into 4 Hz warrants the Nyquist frequency be well above the highest respiratory frequency.

Subsequently, any frequency- or time-frequency-based analysis can be applied to estimate the respiratory frequency. In most studies the average estimated RR has been obtained by windowing the derived modulation and performing spectral analysis to obtain an averaged respiratory frequency. Here, we propose to apply two methods: either an AR spectral model or use of the Hilbert spectrum to estimate average respiratory frequency and calculating average RR in each window.

Then, we have applied the WSST to find the time-frequency spectrum of the derived AM or FM signal where the given spectrum is masked using average RRs. The IRRs are then estimated at each time point based on frequencies with maximum energy in the WSST where SSWT(r(t)) has been calculated by first replacing s(t) by r(t) (respiratory modulation) in Equation (1) to obtain $W_{r}\left(a,b\right)$ and then applying steps 2–3 to implement Equations (3) and (4) to obtain $T_{r}\left(\omega_{l},b\right)$ that is represented by $W_{ssw}$ in Algorithm 1. For our dataset which includes healthy subjects, we achieved good results using either FM or AM signals. For studies involving patients, the use of FM signals might not produce such reliable results, noting the diminishing effect of respiratory sinus arrythmia with age [39].

### 2.6. Dataset

In this paper two datasets are used to apply the proposed method for estimation of instantaneous respiratory rate. For both datasets, the PPG signals were recorded from a selected body position and the thermocouple was used to provide the reference data for breath-by-breath analysis. The thermocouple was secured under the nose to be able to detect the warm air flowing out of the nostrils at each expiration. The generated signal has been used to estimate reference respiratory rate.

For the first dataset, the PPG signals were recorded using a custom device. The customised device was based on an Arduino UNO board connected to a probe with a MAXREFDES117# reflectance PPG sensor and the Cooking Hacks e-Health Sensor Platform V1 to connect the thermocouple. The PPG sensor samples red and infrared signals at 100 Hz.

The signals were recorded at various body position as shown in Figure 1 from five healthy male subjects, all physically and mentally fit and aged between 30 and 40. The subjects were sitting comfortably in an armchair facing a computer monitor in an office room. The body positions include forehead, ear, nose, neck, chest, arm, finger and wrist. The PPG signals were recorded from the wrist in three places including head of the ulna (noted as wrist bone), wrist centers visible in dorsal and palmar views. All the data were transferred on a serial-emulated connection over USB to a MATLAB script that stored the signals on comma-separated files.

In the second dataset, the signals were recorded using a commercial wrist-worn PPG sensor (WaveletHealth Inc., Mountain View, CA, USA).This wristband records two-wavelength PPG signals using red and infrared lights where the sampling frequency was fixed at 86 Hz. The data was then transferred to a mobile phone app and made available for download from the company’s web server. One subject wearing the wavelethealth sensor and the thermocouple is shown in Figure 2. Another set of five healthy subjects (3 female, 2 male) aged between 25 and 35, all physically and mentally fit, participated in this second round of experiments. For this dataset, the subjects were also sitting comfortably in an armchair facing a computer monitor in an office room. Most subjects voluntarily had few continuous deep breaths in the experiment to induce some variations in their breathing’s pattern. Both datasets included healthy subjects where the ethics approval was obtained prior to performing experiments.

## 3. Results

For the first dataset, IRR has been estimated from infrared PPG signals recorded using the custom device from various body positions where simultaneous breathing signal was recorded using the thermocouple. For each of the 5 subjects, we have selected five body positions where a high correlation between the estimated IRR and reference data was obtained. For every location, an average error between reference and estimated IRRs has been calculated, then locations with less errors are selected. To calculate the average error, first, RRs per 100 s (one window) are averaged for the reference RRs and IRRs, separately.

**Figure 4:**
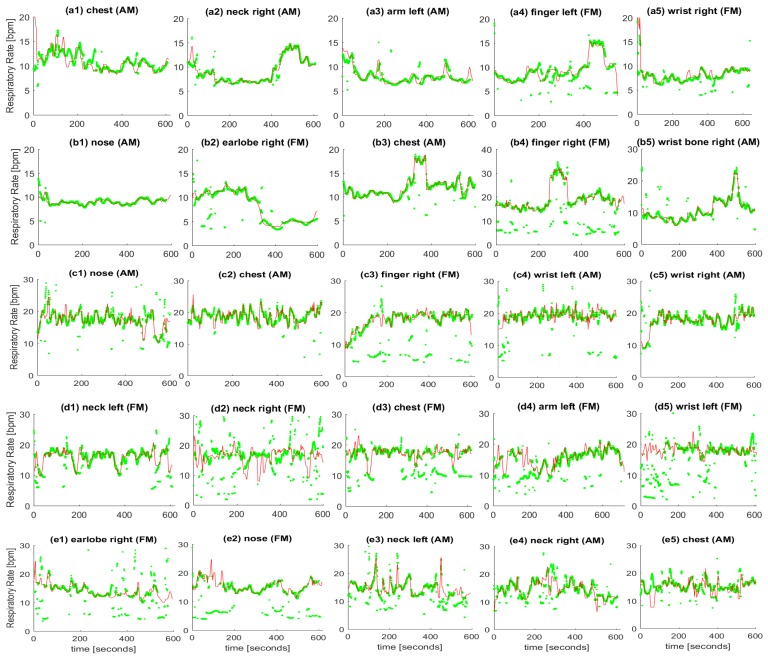
Reference respiratory rate (solid line with red color) and estimated instantaneous respiratory rate using the proposed method (green dots) from selected body position and respiratory induced modulation for subject #1 (**a1**–**a5**), subject #2 (**b1**–**b5**), subject #3 (**c1**–**c5**), subject #4 (**d1**–**d5**), subject #5 (**e1**–**e5**).

Then, considering all the windows, the mean absolute errors (between averaged reference RRs and IRRs in each window) are obtained. Based on this calculated average error metric, the top five locations for our dataset are selected. These selected locations are shown in Figure 4 ((a1–a5) for subject #1, (b1–b5) for subject #2, (c1–c5) for subject #3, (d1–d5) for subject #4, (e1–e5) for subject #5). For these subjects, the AM or FM signals were selected based on the smoothness of the resulted time-frequency spectrum given by the WSST. The mean absolute errors (MAEs) explained above are calculated and provided in Table 1. For each table entry (a selected subject considering one specific sensor location), the corresponding plot is provided in Figure 4.

It is worth noting that the plots shown in Figure 4 and their calculated MAEs in Table 1 are not sorted based on the calculated average errors between estimated IRRs and reference RRs for each subject, however, from left to right, the locations tend towards the lower body parts. For example, earlobe/nose locations have appeared in the first/second columns while wrist location has appeared in the last column for most subjects (see Table 1). The lowest recorded MAE (0.13) relates to subject #2 from nose while the second lowest recorded MAE (0.22) relates to subject #3 from wrist. Overall, the results of estimated IRRs are promising for most body positions. For some subjects in certain locations sparsely invalid estimated IRR can be observed, which can be removed by applying further frequency-tracking based filtering techniques. From the results, a single best location has not been found for various subjects noting that the PPG signal was recorded from only one position at a time.

However, the chest has consistently provided a good estimate of IRR for almost all subjects as appeared for every subject in Table 1. The wrist ulna head as a new location, appeared among top locations for the subject #2 which has been noted as wrist bone in Table 1 and the corresponding plot title in Figure 4. The results from wrist front (dorsal view) noted as wrist in Figure 4 are promising for subject #1 and subject #3. This position is considered as the main location for recording the PPG signals when using the smart watches or smart wearable wristbands.

Based on analysis of the estimated IRR from various body positions, our algorithm has shown to be capable of tracking changes in the respiratory rate.

In the second dataset, the PPG signals recorded from a commercial were analysed where samples of IRR are compared to the averaged estimates of RR. As shown in Figure 2, the subjects were equipped with the WaveletHealth wristband. A simultaneous accelerometer is available in the wristband which could be useful to perform joint motion and breath-by-breath analysis. The simultaneous thermocouple also shown in Figure 2 to generate the reference data for breath-by-breath analysis used at sampling frequency of 100 Hz. In the second dataset, five healthy subjects participated in the experiment have various skin colors.

**Figure 5:**
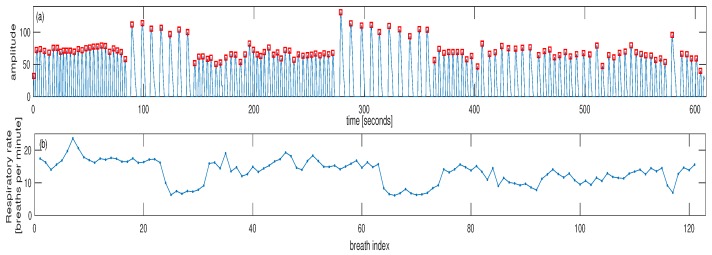
(**a**) The generated signal using thermocouple from subject #1, each pulse is related to one breath. (**b**) Estimated individual breaths using the timing between pulse peaks. There are two episodes of low breathing for this subject. The pulse peaks are noted by red squares in the top plot.

**Figure 6:**
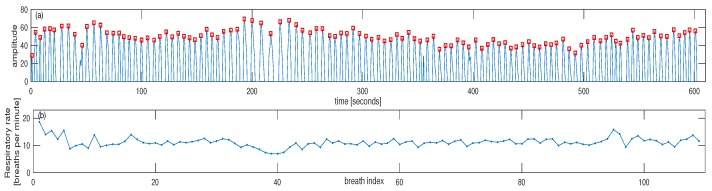
(**a**) The generated signal using thermocouple from subject #3, each pulse is related to one breath. (**b**) Estimated individual breaths using the timing between pulse peaks. There is one main episode of low breathing for this subject. The pulse peaks are noted by red squares in the top plot.

**Figure 7:**
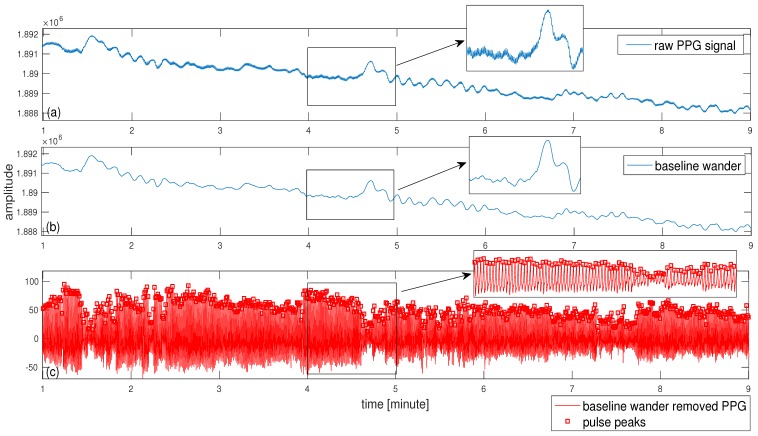
(**a**) Raw PPG signal (infrared); (**b**) Baseline wander that is removed from raw PPG signal in (**a**); (**c**) Separated pulse signals by subtraction of baseline wander from (**a**) using SSA algorithm (see Algorithm 1). Detected pulse are located into the baseline wander removed PPG.

For the second dataset, the signal generated from the thermocouple is shown in Figure 5a for subject #1 where the peaks are detected to denote each breath. Based on the timing between consecutive peaks, the estimated IRR could be calculated in terms of breaths per minute. This is shown in Figure 5b for subject #1. As it can be seen from Figure 5, there are two episodes of low breathing rate for this subject. As another example, the reference signals and estimated IRR are shown in Figure 6 for the subject #3. For this subject, there is one episode with a small decrease in the breathing rate at around 40th breaths (Figure 6b). The IRR is estimated from infrared PPG which demonstrated a better quality over red PPG. An example of raw infrared PPG is shown in Figure 7a. The SSA algorithm with an input embedding dimension of M=50 and raw PPG has been applied. In the first step of the SSA, the trajectory matrix is constructed by delaying the input raw PPG signal (M-1 times) [32].

**Figure 8:**
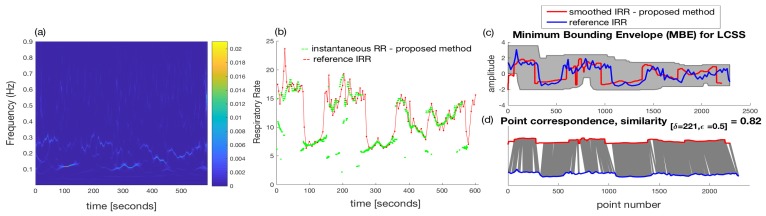
(**a**) The wavelet synchrnosqueezed transform of the derived and resampled FM for subject #1; (**b**) Estimated instantaneous respiratory rate using the proposed technique. The reference is shown in red color; (**c**,**d**) Similarity of estimated instantaneous respiratory rate and the reference using the longest common subsequence algorithm. A similarity of 0.82 (maximum similarity is 1) has been obtained for subject #1. The reference RR has been shown in blue color in (**c**,**d**).

**Figure 9:**
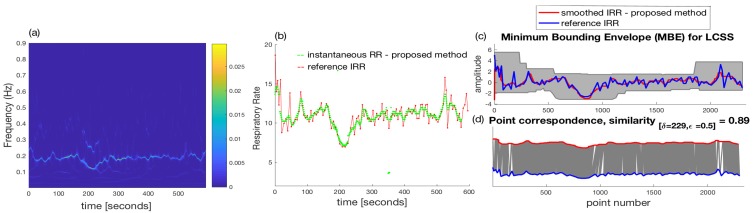
(**a**) The wavelet synchrnosqueezed transform of the derived and resampled FM for subject #3; (**b**) Estimated instantaneous respiratory rate using the proposed technique. The reference is shown in red color; (**c**,**d**) Similarity of estimated instantaneous respiratory rate and the reference using the longest common subsequence algorithm. A high similarity of 0.89 (maximum similarity is 1) has been obtained for subject #3. The reference RR has been shown in blue color in (**c**,**d**).

After applying the SVD to the resulted trajectory matrix, as an example, the eigenvectors ($u_{1},v_{1}$) with the largest eigenvalue ($\lambda_{1}$) which correspond to the trend of the raw PPG can be used to reconstruct the trend as shown in Figure 7b ($\left[\sqrt{\lambda_{1}}u_{1}v_{1}^{T}\right]$). This trend has been separated from the raw PPG including removal of high frequency noises to produce the signal shown in Figure 7c by using a set of indices ($\hat{X}=\sum_{j=2,3,...,L=40}\left[\sqrt{\lambda_{j}}u_{j}v_{j}^{T}\right]$) in the SSA algorithm to be used for signal reconstruction. This implies that the eigenvectors relating to the largest eigenvalue presenting the baseline wander (*j* = 1) and very small eigenvalues (*j* = 41, ..., *M* = 50) are excluded to reconstruct PPG signal shown in Figure 7c (see Algorithm 1). Then, peakdet algorithm has been applied to this signal using peakdet function (Matlab, the MathWorks Inc., Natick, MA, USA). After detection of pulse peaks, respiratory induced modulation needs to be extracted and resampled into a 4 Hz signal. We empirically found frequency modulation signals to provide a better time-frequency representation than other modulations for our dataset. Following estimation of average RRs, WSST has been applied to respiratory modulation where masking in time and frequency domain has been also performed. Average RRs estimated for each time-window are used to find an appropriate frequency regions in the WSST transform to help extraction of valid instantaneous frequencies. For masking, 32 s time-windows (δt in Algorithm 1) and frequency windows with 0.3 Hz length (≈2ω in Algorithm 1) are used to find maximum energy of the SSWT spectrum at each time point and locate the corresponding frequency. Selection of larger windows e.g., 64 s could result in a lower average error than the use of 32 s. However, the algorithm may not be able to respond to faster changes in the RRs when using a larger window size. For subject #1 and subject #3, the time-frequency representations of the extracted FM, reference breathing rate and estimated IRR are shown in Figure 8a,b and Figure 9a,b.

Since the WSST has been applied to the derived frequency modulation re-sampled at 4 Hz, the IRR is estimated each 0.25 s. The duration of the recordings lasted for almost 10 min (600 s). Therefore, almost 2400 points are obtained by using the instantaneous time-frequency pairs using the proposed method.

The number of estimated IRR points using the reference data is much less than the points obtained using a 4 Hz re-sampled modulations. This is because the IRR estimations using the reference are related to breath-by-breath variation; one sample of IRR is estimated per one breath. To quantify the results, using the estimated IRR including the actual timing of each breath, the reference IRR is re-sampled into a 4 Hz signal using interpolation.

Then, longest common subsequence algorithm (LCSS) [40,41] was used to find the similarity of between reference IRR and estimated IRR using the proposed algorithm. The LCSS algorithm uses dynamic programming to measure the similarity of two time-series with different lengths by matching points based on their distances. One advantage of the LCSS algorithm is the ability to set matching regions in time and space by the input parameters to prevent matching points in regions that are distant or degenerate. The reason to use the LCSS for measuring the similarities was slight displacements in the actual timings of breaths in the reference signals after re-sampling; this is taken into account by matching the points in specified time-space region using the LCSS algorithm.

The results are plotted in Figure 8c,d and Figure 9c,d for subject #1 and subject #3, respectively. A high similarity of 0.89 (maximum similarity is one) LCSS has been obtained for the subject #3.

**Figure 10:**
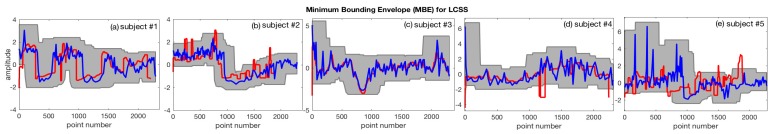
Estimated instantaneous respiratory rate for five subjects wearing the Wavelethealth sensor (red curve) versus reference respiratory rate obtained using thermocouple (blue curve): (**a**) subject #1; (**b**) subject #2; (**c**) subject #3; (**d**) subject #4; (**e**) subject #5.

The smoothed IRR signal using our proposed method versus the reference IRR is shown in Figure 10 for all the five subjects in the second dataset. The similarities of the reference and calculated IRR are obtained as 0.82, 0.58, 0.89, 0.77 and 0.41 for subjects #1, 2, 3, 4 and 5, respectively.

**Figure 11:**
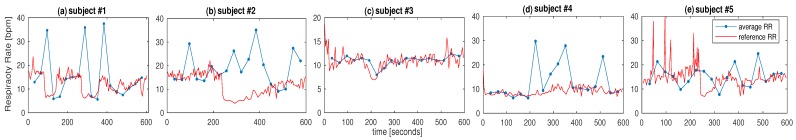
Estimated average instantaneous respiratory rate using the AR-spectral based method for five subjects wearing the Wavelethealth sensor. Fewer number points (18 or 19) have been obtained using windows of 32 s long. There is a mis-agreement for many segments: (**a**) subject #1; (**b**) subject #2; (**c**) subject #3; (**d**) subject #4; (**e**) subject #5.

The windowed segments of PPG with 32 s long (with zero overlap) are used to estimate average respiratory rate using the AR spectral method. The estimated RR is plotted in Figure 11. About 18–20 data points are produced for each subject and deviations from the reference IRR could be easily seen (Figure 11, subject #1, #2 and #3). This also resulted in very low similarity values in the LCSS algorithm for most subjects due to a larger difference in the lengths of reference RR and averaged RR using windows with zero overlap. In addition, there is a huge difference between the number of RRs when comparing average RRs for 32 s window with zero overlap (≈19 points for 600 s) to IRRs which are estimated from a 4 Hz signal instantaneously (2400 points for 600 s).

Although our method resulted into sparse estimated RR samples, they could be removed by developing a frequency tracking filtering based technique. We also increased the overlap between segmented windows of 32 s length into 28 s overlap. The similarities of the reference and calculated average RR using AR spectral method are obtained as 0.52, 0.33, 0.51, 0.41 and 0.68 for subjects #1, 2, 3, 4 and 5, respectively. Comparing these similarity values to those obtained by our proposed algorithm, except for the subject #5 which sparse estimated RRs affected the smoothed IRRs, our method outperforms the averagely estimated RRs. By more processing and denoising of WSST, improved estimations are expected for IRRs. Overall, calculated similarity values quantitatively confirms the superiority of our proposed algorithm in estimation of IRR.

## 4. Discussion

In this paper a new approach is proposed to estimate instantaneous respiratory rate from reflectance PPG signals incorporating wavelet synchrosqueezed transform. A custom device and a commercial wearable wristband were used to collect PPG data. A simultaneous thermocouple signal was used to generate the reference data for breath-by-breath analysis.

The WaveletHealth wristband was used in recent research studies to detect arterial fibrillation (AF) from wrist PPGs with an accuracy of 91% where ECG based Xio Patch (iRhythm Tech. Inc., San Francisco, CA, USA) was used as the ground truth [42].In another study, the sensor has also been used as a portable screening device for obstructive sleep apnea and evaluated versus polysomnography (PSG) system for estimation of the heart rate, heart rate variability, blood oxygen level (SpO${}_{2}$) and RR parameters [43]. In a study on patients with obstructive Hypertrophic cardiomyopathy (OHCM) using WaveletHealth wristband [44], it has been found that the combination of a wrist-worn biosensor and associated machine learning algorithms can be used to identify a signature of arterial blood flow in OHCM patients compared to unaffected controls. The findings are important to non-invasively detect OHCM. The sensor has also been recently used to estimate blood oxygen level in comparison with other commercial sensors [45].

WaveletHealth sensor as a small wristband has been validated to unobtrusively generate clinical-grade PPG signals as suggested in above clinical studies. Our new platform which has validated estimation of IRR using WaveletHealth sensor opens a new area into sleep analysis application based on long-term monitoring of IRR and observing variations in estimated respiratory rate. We will be able to observe the joint time-frequency of the re-sampled modulations in continuous long-term recordings. This can provide new insights into variability of respirations over various sleep stages. Based on the results, instantaneous frequency variations in the joint time-frequency spectrum highly correlated with instantaneous respiration have been observed. This will have a significant application for improving sleep monitoring systems to observe variations in RR over a long period of time. Moreover, visualisation of joint time-frequency spectrum of respiratory modulations for a long period can be used to identify variations of RRs over time that may help clinicians detect crucial changes in the RRs to predict critical situations. The proposed platform can be used to continuously monitor patients in the ICUs and be able to prevent patient deteriorations based on observed variations of RR over time.

In future studies, motion affected PPG signals can be recorded to further improve the proposed method, including the analysis of simultaneous acceleration signals. In addition, frequency tracking with the aid of other filtering techniques such as Kalman smoothing can be applied to the WSST spectrum in future studies to remove the sparse invalid estimated IRRs for an improved performance.

Evaluation of PPG signals recorded from various body positions can be studied comprehensively in future studies. The objective of this paper was to examine PPG signals recorded for estimation of IRR from multiple body positions versus the recorded reference data. The dataset lacked simultaneous PPG recordings for a proper comparison of various body positions. Nonetheless, chest has proved to be able to be consistently in top selected five positions for all the subjects. In general, upper body positions produced PPG signals with a higher quality than for example other positions such as finger or wrist. While places like forehead and finger have been analysed in various research studies, other body positions such as wrist bone (ulna head) and front/back of wrist are less investigated for estimation of respiratory rate. These positions can be further evaluated.

In future studies, long-term recordings of the PPG data from patients should be performed. One challenge in the inclusion of patients data in large clinical trials to further improve and evaluate the proposed method for estimation of IRR, is the reliability of the reference data. A good candidate to estimate reference RR and generate ground truth data in the clinical environment will be the use of other commercial sensors such as wearable ECG patches clinically validated for estimation of respiratory rate. Optical based sensors are unobtrusive ways to record PPG signals that would be used to estimate IRR, while ECG patches as less unobtrusive ways can be used to validate estimation of IRR from a wearable sensor specifically WaveletHealth wristband in future studies.

## 5. Conclusions

In this research, breath-by-breath analysis of respiration using a developed algorithm from reflectance PPG sensors is validated on healthy subjects considering various body positions. Detailed comparison of body positions has been out of the scope of this paper, however, chest has consistently ranked among top five locations with higher calculated accuracies for estimated IRRs. Although wrist is not the best location to record PPG signals due to sensitivity of the optical sensors to wrist movements, it is considered as a convenient location with a high patient compliance. In future studies, data from patient groups should be recorded from wrist to evaluate estimation of IRRs in clinical settings and for long term monitoring where inclusion and assessment of IM are recommended. Long term monitoring of breathing patterns and their variations could provide clinicians with crucial information for certain patients such as those with COPD or inside intensive care units.

## Figures and Tables

**Table 1:** The MAE of estimated IRRs versus reference data from thermocouple using our proposed algorithm in top five selected locations with lowest errors in the first dataset (Figure 4).

Subject ID	Error (Location)				
#1	0.69 (chest)	0.72 (neck-R)	0.25 (arm-L)	0.43 (finger-L)	0.66 (wrist-R)
#2	0.13 (nose)	0.36 (earlobe-R)	0.45 (chest)	3.76 (finger-R)	0.67 (wrist-bone-R)
#3	0.44 (nose)	0.31 (chest)	2.65 (finger-R)	1.47 (wrist-L)	0.22 (wrist-R)
#4	1.17 (neck-L)	4.41 (neck-R)	2.18 (chest)	3.39 (arm-L)	4.61 (wrist-L)
#5	2.31 (earlobe-R)	5.20 (nose)	1.65 (neck-L)	0.52 (neck-R)	0.49 (chest)
